# Alcohol‐Induced Blackouts and Subsequent Drinking Trajectories: Evidence From Three Longitudinal Samples

**DOI:** 10.1111/acer.70373

**Published:** 2026-07-20

**Authors:** Angelo M. DiBello, Mary Beth Miller, Kate B. Carey, Clayton Neighbors

**Affiliations:** ^1^ Center for Alcohol and Substance Use Studies & Department of Graduate and Applied Professional Psychology Rutgers University Piscataway New Jersey USA; ^2^ Department of Psychiatry University of Missouri‐Columbia Columbia Missouri USA; ^3^ Department of Behavioral and Social Sciences Brown University School of Public Health Providence Rhode Island USA; ^4^ Department of Psychology University of Houston Houston Texas USA

**Keywords:** alcohol, alcohol‐induced amnesia, alcohol‐related consequences, blackout

## Abstract

**Background:**

Alcohol‐induced blackouts are widely recognized as markers of acute intoxication and elevated risk for alcohol‐related harm. Blackout experiences may also represent psychologically salient events capable of influencing subsequent drinking behavior. The present study examined whether recent blackout predicts changes in alcohol use, alcohol‐related consequences, and blackouts over time.

**Method:**

Data were drawn from three independent longitudinal samples of college student drinkers (*N*s = 568, 484, and 200). Generalized linear mixed models with negative binomial distributions were used to evaluate whether baseline blackout status predicted follow‐up drinks per week, alcohol‐related consequences, and subsequent blackouts over 12 months of follow‐up, controlling for baseline drinking and consequences, age, sex, intervention condition, and time.

**Results:**

Across samples, baseline blackout was not associated with increases in subsequent alcohol use. In one sample, blackout predicted lower follow‐up drinking, and in two of three samples, blackout predicted significantly lower rates of alcohol‐related consequences over time (i.e., a 21%–40% reduction in consequences among those who experienced a blackout across follow‐ups). Conversely, baseline blackout was associated with increased odds of subsequent blackout across all samples. These associations were found after adjusting for baseline alcohol‐related consequences and concurrent drinking.

**Conclusion:**

Blackouts may function not only as a marker of high‐risk drinking but also as a potential turning point associated with reductions in alcohol‐related harm. At the same time, effects were not uniform across samples. Results underscore the importance of assessing blackout in research and clinical settings, and they highlight its potential dual role as both a risk indicator and a catalyst for behavioral change.

## Graphical abstract

Alcohol‐induced blackouts may serve as both a marker of continued risk and a catalyst for change: students who experienced a baseline blackout showed reduced alcohol use in one sample and fewer alcohol‐related consequences in two samples, yet remained at elevated risk for future blackouts across all three samples.

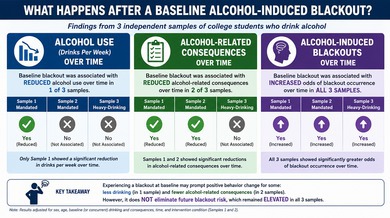

## 1 Introduction

Alcohol‐induced blackouts, defined as periods of anterograde amnesia that occur during episodes of heavy drinking, are among the most serious and concerning outcomes of alcohol use in young adults (Hingson et al. 2016). A blackout occurs when rapid increases in blood alcohol concentration disrupt the transfer of short‐term to long‐term memory, resulting in partial (fragmentary) or complete (en bloc) memory loss for events that occurred while intoxicated (White 2003). Although individuals are awake and engaging with the environment during these episodes (e.g., talking, driving), they are later unable to recall their behavior. Blackouts are alarmingly common: over half of young adult drinkers report experiencing at least one blackout in their lifetime (Hingson et al. 2016; Wetherill and Fromme 2016), with rates ~60% in heavy‐drinking samples (Acuff et al. 2019) and as many as 50%–54% of mandated college students report a blackout in the last month (DiBello et al. 2023). Moreover, blackout episodes tend to recur within individuals over time (Barnett et al. 2014; Merrill et al. 2016), underscoring their relevance as a repeated high‐risk drinking pattern rather than a rare event. Indeed, between 17% and 22% reported two or more blackouts in the previous year (DiBello et al. 2023). In addition to their prevalence, blackouts are associated with a range of adverse consequences including physical injury, sexual victimization, emergency department visits, and later development of alcohol use disorder (Hingson et al. 2016; Wilhite and Fromme 2015). Longitudinal research indicates that blackouts prospectively predict subsequent social and emotional consequences during the transition out of college, even after accounting for heavy drinking patterns (Wilhite and Fromme 2015), underscoring their prognostic significance beyond simple consumption levels.

Despite their prevalence and severity, blackouts may also represent psychologically meaningful events within the drinking trajectory. A blackout is not only a marker of excessive consumption but also a highly salient and memorable experience (ironically defined by its lack of memory) that can evoke shock, embarrassment, fear, or concern among drinkers and their peers. Such reactions may stem in part from the increased risk of alcohol‐related consequences, particularly serious consequences (e.g., legal, sexual), on blackout versus nonblackout drinking days (Merrill et al. 2019; Richards et al. 2023). The manner in which students drink also independently predicts blackout risk, with faster rates of intoxication, higher peak intoxication, and longer rise durations each associated with greater odds of experiencing a blackout (Carpenter and Merrill 2021; Richards et al. 2024). Consistent with this idea, qualitative and event‐level studies have shown that young adults describe blackouts as “wake‐up calls,” prompting self‐reflection or intentions to drink less (Merrill et al. 2019; Miller, Merrill, et al. 2018), and longitudinal studies have linked blackouts to increases in motivation to reduce alcohol use (Marino and Fromme 2018).

Experimental and intervention‐based research also suggests that blackout experiences may function as teachable moments. For example, individuals who have recently experienced blackout demonstrate greater reductions in drinking or consequences following personalized normative feedback compared to those without blackout (Miller et al. 2019; Miller, DiBello, et al. 2018). Similarly, blackout has been shown to moderate the association between drinking attitudes and subsequent behavior, such that individuals who experience blackout and hold less favorable views of heavy drinking show greater reductions in peak intoxication over time (DiBello et al. 2021).

At the same time, blackout itself is not universally perceived as negative (e.g., Mallett et al. 2008; Merrill et al. 2020). A subset of young adults reports intentional drinking to the point of memory loss (Miller et al. 2020), and others remain willing to blackout, even if they do not intend to blackout (Merrill et al. 2022). Indeed, willingness to experience a blackout, reflecting openness to the experience rather than a deliberate plan, has been shown to prospectively predict both blackout frequency and alcohol use disorder (AUD) risk symptoms throughout college (Glenn et al. 2024). Taken together, this research suggests that attitudinal orientation toward blackouts may be as consequential as drinking behavior itself. Thus, the impact of blackout depends in part on how the experience is appraised, with some individuals interpreting it as aversive and others normalizing or even seeking it.

Understanding whether and how blackout experiences influence subsequent drinking trajectories is essential for both theory and intervention. Much of the prior work has conceptualized blackout as either a consequence of heavy alcohol use or as a predictor of future harm. For example, Wilhite and Fromme (2015) demonstrated that blackouts prospectively predicted increases in social and emotional consequences during the transition out of college, even when controlling for drinking quantity. However, few studies have examined whether blackout experiences are associated with changes in alcohol use or alcohol‐related harm over time (Marino and Fromme 2018; Read et al. 2013; Wilhite and Fromme 2015). Because blackouts are often unexpected and emotionally charged, they may function as naturally occurring “behavioral interventions,” motivating some individuals to reevaluate their drinking (e.g., Marino and Fromme 2018). Conversely, repeated blackouts without behavioral adjustment may signal escalating risk and diminished sensitivity to negative consequences (e.g., Yuen et al. 2021). Thus, blackouts may operate in dual ways: as a prognostic indicator of sustained harm and, under certain conditions, as a catalyst for change.

### 1.1 Current Study

The present study addresses this gap by testing whether experiencing an alcohol‐related blackout predicts subsequent change in alcohol use, alcohol‐related consequences, and blackouts over time. The current work sought to examine whether a recent blackout represents a significant predictor of change in drinking and alcohol‐related consequence trajectories across three independent samples. Specifically, we test whether individuals who report a recent blackout at baseline evidence differences in alcohol use, associated consequences, and blackout relative to those who do not. We aimed to isolate the effect of blackout by controlling for contemporaneous baseline use and consequences. This approach allows for a nuanced understanding of blackouts not merely as outcomes of risky drinking but as a potential catalyst for behavior change among heavy‐drinking young adults.

## 2 Method

### 2.1 Participants and Procedures

#### 2.1.1 Sample 1

Undergraduate students from a large public university in the Northeastern United States were recruited on a rolling basis between 2011 and 2013 to participate in a larger research study evaluating the efficacy of an intervention for students mandated for campus alcohol violations (Carey et al. 2018). All participants received the same brief alcohol intervention after baseline, which resulted in reductions in alcohol use and consequences at the 1‐month assessment (Carey et al. 2018). Immediately following the 1‐month follow‐up, participants received a series of 12 email boosters, containing either corrective alcohol norms or general health information, based on random assignment. Follow‐up assessments were completed at 3, 5, 8, and 12 months. The RCT portion of the study did not result in group differences on outcomes (Carey et al. 2018). The baseline and 1‐month assessments consisted of online surveys completed in a private suite and facilitated by a research assistant, whereas all other assessments were completed remotely. All students who consented to participate in the larger study were included in current analyses.

The final sample consisted of 568 students (71.7% male; 84.0% White) with a mean age of 19.18 years (SD = 1.16). At baseline, participants reported drinking a mean of 12.6 (SD = 9.8) drinks per week, 5.64 (SD = 4.39) total consequences in the past month, and 44.42% reported a blackout in the past month. All study procedures were approved by the university's Institutional Review Board.

#### 2.1.2 Sample 2

Participants were college students from a different public university in the Northeastern United States who had been cited for violating a campus alcohol use policy and referred to the Office of Alcohol, Tobacco, and Other Drugs (ATOD). All students mandated to participate in an alcohol intervention at ATOD were screened by a trained research assistant to determine eligibility for participation in the parent study (Carey et al. 2024). Participants who consented completed a baseline assessment, were randomized to either a control or self‐affirmation writing activity, completed the eCHECKUP TO GO intervention (Moyer et al. 2004), and completed a postintervention assessment within the same session. Participants assigned to the control writing activity listed foods consumed in the previous 24 h and wrote a description of the first item listed. Participants assigned to the self‐affirmation writing activity listed their five most important values in order of importance and wrote about their highest‐ranked value and how they had expressed it in their lives. Follow‐up surveys were completed remotely at 1, 3, 6, 9, and 12 months. All students who consented to participate in the larger study were included in current analyses.

The sample included 484 individuals (M_age_ = 18.66 years, SD = 0.76; 55.6% male). Participants identified as 77.6% White, 11.8% Asian, 3.7% Black/African American, 0.6% Native Hawaiian/Pacific Islander, 0.2% American Indian/Alaska Native, and 6.0% another race; 11.6% identified as Hispanic/Latino. At baseline, participants reported consuming a mean of 8.90 (SD = 7.74) drinks per week, 4.64 (SD = 4.39) total consequences in the past month, and 33.26% reported a blackout in the past month. All study procedures were approved by the university's Institutional Review Board.

#### 2.1.3 Sample 3

Participants were enrolled in a randomized controlled trial designed to examine a novel cognitive dissonance‐based brief intervention for heavy‐drinking college students at two universities (Carey et al. 2025). Campus 1 was a large public university in the Southern United States, and Campus 2 was a medium‐sized private university in the Northeastern United States. The experimental conditions of the parent study included an assessment‐only control condition, a gender‐specific personalized normative feedback (PNF) condition, and a counter‐attitudinal advocacy (CAA) condition. The study utilized a longitudinal design consisting of an in‐person or video‐conference baseline session and three follow‐up assessments at 1, 3, and 6 months postbaseline. Of the 590 who were enrolled and randomized at baseline (control = 200; CAA = 194; PNF = 197), the present study used data from the 200 participants assigned to the assessment‐only control condition and their 1‐, 3‐, and 6‐month follow‐up data.

The 200 participants in the assessment‐only control condition were 63.0% female, with a mean age of 20.01 years (SD = 1.67). Participants identified as 52.0% White, 24.0% Asian, 11.5% Black, 2.0% Native American/Alaska Native, and 10.0% another race; 23.0% identified as Hispanic/Latino. With respect to campus location, 38.0% were enrolled at Campus 1 and 62.0% at Campus 2. At baseline, participants reported consuming a mean of 10.06 (SD = 7.833) drinks per week, 11.54 (SD = 7.20) total consequences in the past month, and 28.0% reported a blackout in the past month. All study procedures were approved by the Institutional Review Boards of the participating institutions.

### 2.2 Measures

#### 2.2.1 Demographic Characteristics

Across all three studies, participants reported their age and sex assigned (0 = female, 1 = male) at birth at baseline.

#### 2.2.2 Alcohol Consumption

In each sample, alcohol use over the past month was measured using a 7‐day grid that captured the typical number of drinks consumed on each day of a usual week. This format was adapted from the Daily Drinking Questionnaire (Collins et al. 1985). A standard drink was defined as a 12‐oz beer, a 5‐oz serving of wine, or 1.5 oz of distilled liquor. Weekly alcohol use was calculated by summing the total number of standard drinks reported across the 7 days.

#### 2.2.3 Alcohol‐Related Consequences

In all three studies, past‐month drinking consequences were assessed using items from the Young Adult Alcohol Consequences Questionnaire (YAACQ; Read et al. 2006). Studies 1 and 2 used the brief version of the scale (BYAACQ; Kahler et al. 2005), a 24‐item self‐report checklist assessing alcohol‐related problems, with yes/no responses indicating whether each consequence occurred in the previous month. Study 3 used the full 48‐item YAACQ. Sample items include experiencing nausea or vomiting after drinking and waking up in an unfamiliar location following heavy alcohol use. For all samples, consequence scores were computed by summing endorsed items, with the blackout item excluded from the total score.

#### 2.2.4 Primary Predictor

##### 2.2.4.1 Blackout Experience

In all three samples, blackout occurrence during the past month was assessed using a single yes/no item drawn from the YAACQ (Read et al. 2006). Specifically, participants indicated whether they had “not been able to remember large stretches of time while drinking heavily” (0 = no, 1 = yes), which maps onto the en bloc (permanent) form of blackout in factor analytic studies (Boness et al. 2022; Miller et al. 2019).

### 2.3 Plan of Analyses

Longitudinal analyses were conducted using generalized linear mixed models (GLMMs; Atkins et al. 2013; Hedeker and Gibbons 2006) estimated in SAS (PROC GLIMMIX) to examine changes in alcohol use and alcohol‐related consequences over time as a function of blackout status (i.e., having experienced a blackout in the month before baseline), while blackout experiences were modeled as a binary outcome (0 = no, 1 = yes) using multilevel logistic regression. Because weekly alcohol use and alcohol‐related consequences were positively skewed count outcomes, models specified a negative binomial distribution with a log link function. Fixed effects were exponentiated to yield incidence rate ratios for interpretability. Random intercepts were included to account for clustering of repeated observations within individuals.

Analyses focused on post‐baseline change in alcohol use, alcohol‐related consequences, and blackout. Baseline observations for both alcohol use and alcohol‐related consequences were used to construct time‐invariant covariates and were not included as repeated outcome observations in the longitudinal models. This approach ensured that the models estimated change in follow‐up drinking, consequences, and blackout independent of baseline status, rather than modeling baseline as part of the repeated outcome structure.

Follow‐up drinks per week were modeled as a function of blackout status, baseline drinking, baseline consequences, intervention condition (for Samples 1 and 2), sex, age, and time since the first follow‐up assessment. Follow‐up consequences were modeled as a function of blackout status, concurrent drinking, baseline consequences, intervention condition (for Samples 1 and 2), sex, age, and time since the first follow‐up assessment. Follow‐up blackout was modeled as a function of blackout status, concurrent drinking, baseline consequences, intervention condition (for Samples 1 and 2), sex, age, and time since the first follow‐up assessment. This modeling strategy allowed for examination of whether the baseline blackout predictor was associated with changes in outcomes over time, above and beyond initial severity of drinking and consequences. Time was parameterized such that Time = 0 represented the first follow‐up assessment, thereby modeling change following baseline while controlling for initial levels of the outcome. Follow‐up assessments occurred at 1, 3, 5, 8, and 12 months in Sample 1; at 1, 3, 6, 9, and 12 months in Sample 2; and at 1, 3, and 6 months in Sample 3. In each sample, time was treated as a continuous variable representing months since the first follow‐up assessment.

## 3 Results

### 3.1 Sample 1

#### 3.1.1 Alcohol Use

Full model results for all alcohol use models across samples are presented in Table 1. In Sample 1, baseline blackout significantly predicted follow‐up drinks per week. Specifically, individuals who reported a blackout at baseline exhibited lower rates of weekly alcohol use across all follow‐ups compared to those without a blackout (IRR = 0.85, *p* = 0.027). That is, baseline blackout was associated with a 15% reduction in drinks per week during follow‐up.

**TABLE 1:** Drinks per week as a function of baseline blackout.

Outcome	Predictor	Estimate	95% confidence interval	
Drinks per week		IRR	LL95%	UL95%
Sample 1: mandated college students	**Male**	**1.56**	**1.36**	**1.79**
	Age	0.99	0.94	1.04
	Condition	0.93	0.83	1.05
	**Baseline drinks**	**1.05**	**1.04**	**1.06**
	Baseline consequences	0.99	0.98	1.02
	**Baseline blackout**	**0.85**	**0.74**	**0.98**
	**Time**	**1.02**	**1.01**	**1.03**
Sample 2: mandated college students	Male	1.09	0.90	1.31
	**Age**	**1.19**	**1.05**	**1.35**
	Condition	0.99	0.83	1.2
	**Baseline drinks**	**1.08**	**1.07**	**1.1**
	Baseline consequences	1.02	0.98	1.06
	Baseline blackout	0.98	0.75	1.29
	**Time**	**1.02**	**1.01**	**1.03**
Sample 3: heavy‐drinking college students	**Male**	**1.28**	**1.06**	**1.54**
	Age	1.03	0.97	1.09
	**Baseline drinks**	**1.05**	**1.03**	**1.06**
	Baseline consequences	1.01	0.99	1.03
	Baseline blackout	1.05	0.83	1.34
	Time	0.98	0.95	1.01

*Note:* Significant findings in bold. Male coded 0, female; 1, male.

Abbreviations: IRR, incidence rate ratio; LL95%, lower limit of the 95% confidence interval; UL95%, upper limit of the 95% confidence interval.

Time was positively associated with drinks per week (IRR = 1.02, *p* < 0.001), corresponding to a 1.8% increase in drinking with each month of follow‐up. Baseline drinks per week predicted follow‐up drinking (IRR = 1.05, *p* < 0.001), such that each additional baseline drink per week was associated with a 5.1% increase in follow‐up drinking. Sex was also significant (IRR = 1.56, *p* < 0.001), indicating that males reported 56% higher drinking rates than females. Age, intervention condition, and baseline consequences were not significant predictors.

#### 3.1.2 Alcohol‐Related Consequences

Full model results for all alcohol‐related consequence models across samples are presented in Table 2. Baseline blackout significantly predicted follow‐up consequences (IRR = 0.79, *p* = 0.017). Individuals who experienced a blackout at baseline had 21% lower rates of alcohol‐related consequences across all months of follow‐up than those who did not report a blackout at baseline after adjusting for covariates.

**TABLE 2:** Alcohol‐related consequences as a function of baseline blackout.

Outcome	Predictor	Estimate	95% confidence interval	
Alcohol‐related consequences		IRR	Lower	Upper
Sample 1: mandated college students	Male	0.96	0.80	1.15
	Age	0.95	0.88	1.01
	Condition	0.96	0.82	1.13
	**Concurrent drinks**	**1.06**	**1.05**	**1.07**
	**Baseline consequences**	**1.12**	**1.1**	**1.15**
	**Baseline blackout**	**0.79**	**0.65**	**0.96**
	**Time**	**1.02**	**1.01**	**1.04**
Sample 2: mandated college students	**Male**	**0.67**	**0.53**	**0.85**
	Age	0.99	0.84	1.16
	Condition	1.22	0.96	1.55
	**Concurrent drinks**	**1.08**	**1.06**	**1.09**
	**Baseline consequences**	**1.19**	**1.15**	**1.24**
	**Baseline blackout**	**0.60**	**0.42**	**0.85**
	**Time**	**0.98**	**0.96**	**0.99**
Sample 3: heavy‐drinking college students	Male	0.84	0.69	1.03
	Age	0.99	0.93	1.05
	**Concurrent drinks**	**1.07**	**1.05**	**1.08**
	**Baseline consequences**	**1.07**	**1.06**	**1.09**
	Baseline blackout	0.87	0.68	1.12
	Time	0.98	0.95	1.02

*Note:* Significant findings in bold. Male coded 0, female; 1, male.

Abbreviations: IRR, incidence rate ratio; LL95%, lower limit of the 95% confidence interval; UL95%, upper limit of the 95% confidence interval.

Time was positively associated with consequences (IRR = 1.035, *p* < 0.001), reflecting a 3.5% increase in rate of consequences each month. Concurrent drinks per week were strongly associated with consequences (IRR = 1.06, *p* < 0.001), corresponding to a 6.1% increase in consequences per additional drink per week. Baseline consequences were also a robust predictor (IRR = 1.12, *p* < 0.001), indicating a 12.4% increase in follow‐up consequences per additional baseline consequence. Sex, age, and intervention condition were not significant.

#### 3.1.3 Alcohol‐Induced Blackouts

Full results for all the blackout models are presented in Table 3. Baseline blackout significantly predicted follow‐up blackout occurrence (OR = 1.65, *p* = 0.027). Individuals who experienced a blackout at baseline had 64.7% greater odds of experiencing a blackout across follow‐up compared to those who did not report a blackout at baseline after adjusting for covariates.

**TABLE 3:** Blackout as a function of baseline blackout.

Outcome	Predictor	Estimate	95% confidence interval	
Alcohol‐induced blackout		OR	Lower	Upper
Sample 1: mandated college students	Male	0.77	0.47	1.27
	**Age**	**0.79**	**0.65**	**0.98**
	Condition	1.19	0.77	1.86
	Concurrent drinks	1.01	0.98	1.03
	**Concurrent consequences**	**1.58**	**1.47**	**1.69**
	**Baseline blackout**	**1.65**	**1.06**	**2.57**
	Time	1.02	0.99	1.06
Sample 2: mandated college students	Male	0.99	0.48	2.08
	Age	0.87	0.52	1.44
	Condition	0.53	0.25	1.10
	**Concurrent drinks**	**1.07**	**1.03**	**1.12**
	**Concurrent consequences**	**1.98**	**1.72**	**2.27**
	**Baseline blackout**	**2.84**	**1.35**	**5.97**
	Time	0.98	0.92	1.04
Sample 3: heavy‐drinking college students	Male	0.97	0.44	2.18
	Age	0.89	0.72	1.12
	**Concurrent drinks**	**1.05**	**0.99**	**1.11**
	**Concurrent consequences**	**1.25**	**1.16**	**1.34**
	**Baseline blackout**	**3.84**	**1.69**	**8.74**
	Time	0.95	0.81	1.10

*Note:* Significant findings in bold. Male coded 0 = female, 1 = male.

Abbreviations: LL95%, lower limit of the 95% confidence interval; OR, odds ratio; UL95%, upper limit of the 95% confidence interval.

Concurrent alcohol‐related consequences were strongly associated with follow‐up blackout occurrence (OR = 1.58, *p* < 0.001), indicating that each additional concurrent consequence was associated with a 57.7% increase in the odds of experiencing a blackout at that same follow‐up time point. Age was significantly associated with lower odds of follow‐up blackout (OR = 0.79, *p* = 0.030), such that older participants had 20.6% lower odds of experiencing a blackout at follow‐up. Time, concurrent drinks per week, intervention condition, and sex were not significant predictors of follow‐up blackout occurrence.

### 3.2 Sample 2

#### 3.2.1 Alcohol Use

In Sample 2, baseline blackout did not significantly predict follow‐up drinks per week (IRR = 0.98, *p* = 0.892). Thus, experiencing a blackout at baseline was not associated with subsequent changes in alcohol use.

Time was positively associated with drinking (IRR = 1.02, *p* = 0.003), indicating a 2% increase in drinks per week with each month of follow‐up. Baseline drinks per week significantly predicted follow‐up drinking (IRR = 1.08, *p* < 0.001), corresponding to an 8.3% increase in follow‐up drinking per additional baseline drink. Age was positively associated with drinking (IRR = 1.19, *p* = 0.005), indicating a 19% increase in drinking per additional year of age. Sex and intervention condition were not significant. Blackout effects did not vary by follow‐up time‐point.

#### 3.2.2 Alcohol‐Induced Blackouts

Baseline blackout significantly predicted follow‐up blackout occurrence (OR = 2.84, *p* = 0.006). Individuals who experienced a blackout at baseline had 183.5% greater odds of experiencing a blackout across follow‐up compared to those who did not report a blackout at baseline, after adjusting for covariates.

Concurrent alcohol‐related consequences were strongly associated with follow‐up blackout occurrence (OR = 1.98, *p* < 0.001), indicating that each additional concurrent consequence was associated with a 97.9% increase in the odds of experiencing a blackout at that same follow‐up time point. Concurrent drinks per week was also a significant predictor (OR = 1.07, *p* = 0.002), such that each additional drink per week was associated with a 7.2% increase in the odds of experiencing a blackout at follow‐up. Intervention condition, sex, age, and time were not significant predictors of follow‐up blackout occurrence.

#### 3.2.3 Alcohol‐Related Consequences

Baseline blackout significantly predicted follow‐up consequences (IRR = 0.60, *p* = 0.004). Individuals with a baseline blackout exhibited 40% lower rates of consequences during follow‐up compared to those without a baseline blackout.

Time was negatively associated with consequences (IRR = 0.98, *p* = 0.004), reflecting a 2.2% decrease in consequences per month. Drinks per week was strongly associated with consequences (IRR = 1.08, *p* < 0.001), corresponding to a 7.6% increase in consequences per additional drink per week. Baseline consequences were also predictive (IRR = 1.19, *p* < 0.001), indicating a 19.3% increase in follow‐up consequences per baseline consequence. Males reported 33% lower rates of consequences than females (IRR = 0.67, *p* = 0.001). Age and intervention condition were not significant.

### 3.3 Sample 3

#### 3.3.1 Alcohol Use

In Sample 3, baseline blackout did not significantly predict follow‐up drinks per week (IRR = 1.05, *p* = 0.679). Time was not significantly associated with drinking (IRR = 0.98, *p* = 0.139). Baseline drinks per week remained a strong predictor of follow‐up drinking (*b* = 0.046, *p* < 0.001), corresponding to a 4.7% increase in follow‐up drinking per additional baseline drink (RR = 1.05). Sex was significant (IRR = 1.28, *p* = 0.012), indicating that males reported 28% higher drinking rates than females. Age and baseline consequences were not significant.

#### 3.3.2 Alcohol‐Related Consequences

Baseline blackout was not a significant predictor of follow‐up consequences (IRR = 0.87, *p* = 0.271). Time was also not significantly associated with consequences (IRR = 0.98, *p* = 0.288). Concurrent drinks per week were strongly associated with consequences (IRR = 1.07, *p* < 0.001), indicating a 6.7% increase in consequences per additional drink per week. Baseline consequences were also predictive (IRR = 1.07, *p* < 0.001), corresponding to a 7.4% increase in follow‐up consequences per baseline consequence. Sex and age were not significant predictors.

#### 3.3.3 Alcohol‐Induced Blackouts

Baseline blackout significantly predicted follow‐up blackout occurrence (OR = 3.84, *p* = 0.001). Individuals who experienced a blackout at baseline had 283.9% greater odds of experiencing a blackout across follow‐up compared to those who did not report a blackout at baseline after adjusting for covariates.

Concurrent alcohol‐related consequences were strongly associated with follow‐up blackout occurrence (OR = 1.25, *p* < 0.001), indicating that each additional concurrent consequence was associated with a 24.5% increase in the odds of experiencing a blackout at that same follow‐up time point. Sex, age, concurrent drinks per week, and time were not significant predictors of follow‐up blackout occurrence.

Across all models, for all outcomes, the blackout effects did not vary by follow‐up timepoint or by biological sex (i.e., the blackout by follow‐up time point interactions and blackout by biological sex interactions were not significant). These were omitted from the larger results for parsimony.

## 4 Discussion

The present study examined whether experiencing an alcohol‐induced blackout at baseline was associated with subsequent decreases in alcohol use, alcohol‐related consequences, and blackouts over time across three independent samples of college students who drink alcohol. Mostly consistent with hypotheses, in at least one sample, young adults who reported a blackout in the past month at baseline reported greater reductions in subsequent alcohol use over 12 months of follow‐up (Sample 1); and in two of the three samples, those reporting a blackout at baseline reported lower rates of alcohol‐related consequences during follow‐up (Sample 1 and 2). However, baseline blackout experience was associated with significantly greater odds of experiencing a blackout at follow‐up across all three samples. Models controlled for concurrent drinking, baseline consequences (or concurrent consequences when modeling blackout), demographic variables, and intervention condition (Samples 1 and 2), suggesting something unique about blackout experience that may impact subsequent drinking behavior; and the heavy‐drinking volunteers in Sample 3 did not have any triggering or “mandated” event that preceded their study participation. Taken together, these findings suggest that blackout may, in some contexts, represent a meaningful event that helps motivate subsequent behavior change, but they do not appear sufficient to eliminate future blackout risk.

Conceptually, these findings align with prior research suggesting that alcohol‐induced blackouts may function as salient events capable of altering drinking trajectories. Although alcohol‐induced blackouts are reliably associated with elevated risk, including injury, victimization, and later alcohol use disorder (Hingson et al. 2016; Studer et al. 2019; Wetherill and Fromme 2016; Wilhite and Fromme 2015), the present findings suggest that their psychological impact may be more significant than a simple marker of severity. From a cognitive dissonance perspective, blackout episodes may create a discrepancy between individuals' self‐concept (e.g., “I am responsible,” “I am in control”) and behaviors enacted during intoxication. When individuals later learn about actions they cannot remember, this mismatch may generate discomfort, motivating efforts to reduce future dissonance. Qualitative accounts indicate that many young adults experience blackout as unsettling or embarrassing, often describing feelings of regret, fear, or loss of control (Merrill et al. 2019; Miller, Merrill, et al. 2018). Such reactions may heighten awareness of vulnerability and prompt reassessment of drinking behavior. Consistent with this interpretation, intervention studies demonstrate that some individuals with recent blackout histories exhibit greater responsiveness to personalized normative feedback (Miller et al. 2019; Miller, DiBello, et al. 2018), suggesting that a blackout experience can increase receptivity to information that challenges prior beliefs about drinking. Moreover, longitudinal work indicates that blackout interacts with drinking attitudes, such that individuals who do not positively evaluate heavy drinking show greater reductions in peak intoxication over time following blackout (DiBello et al. 2021). Notably, this process is unlikely to be universal. Some individuals report intentions to experience blackout (Miller et al. 2020), potentially reducing dissonance and dampening motivation for change. Thus, blackout may function as a dissonance‐inducing event for some drinkers, whereas for others, it may be cognitively reconciled or normalized. Framing blackout within a cognitive dissonance model helps explain why it may sometimes catalyze harm reduction and other times fail to alter trajectories.

Yet the degree to which these behavioral adjustments translate to reduced blackout risk is less clear. Indeed, consistent with prior diary‐based research, baseline blackout experience was a robust predictor of future blackout occurrence across all three independent samples. These findings replicate and extend prior work suggesting that blackout history is among the strongest predictors of future blackout risk (Glenn et al. 2024; Richards et al. 2023, 2024) and carry important implications for the teachable moment hypothesis central to the current work. Specifically, while individuals who experienced a blackout at baseline demonstrated reductions in alcohol‐related consequences in two of three samples, consistent with the notion that blackouts may function as a catalyst for behavior change, these same individuals remained at elevated risk for future blackout occurrence. This pattern suggests that whatever behavior change is prompted by a blackout experience, it may be insufficient to eliminate future blackout risk. One potential explanation for these findings is that young adults do not completely understand *how* to avoid a blackout, in which case their quantity of drinking changes but the manner of drinking (including speed of intoxication, peak intoxication level, and time spent becoming intoxicated) does not. Thus, even reductions in overall consumption may leave underlying drinking topography largely intact, preserving risk for future blackouts. Alternatively, it is possible that certain individuals may be more genetically predisposed to experiencing blackouts, regardless of behavior change (Davis et al. 2019). The concurrent consequences finding, which replicated across all three samples, further suggests that blackouts and other harms tend to cluster together at the same time points, lending support to the argument that consequences co‐occurring with blackouts may be as relevant to understanding drinking trajectories as the blackout experience itself.

These findings have important implications for prevention and intervention efforts on college campuses. First, the results reinforce the importance of routinely assessing alcohol‐induced blackout in screening protocols, as it may provide information not only about risk but also about readiness for change. Blackout is typically treated as an adverse outcome to be prevented; however, when it occurs, it may create a moment of heightened receptivity to intervention. Brief motivational interventions could capitalize on recent blackout experiences by exploring the emotional and cognitive reactions surrounding the event. Given prior evidence that blackout can increase responsiveness to personalized feedback (Miller et al. 2019; Miller, DiBello, et al. 2018), incorporating targeted discussion of blackout into intervention content may enhance effectiveness. Additionally, screening for blackout in campus health settings may help identify students who are at elevated risk but also potentially primed for change. Importantly, not all individuals who experience blackout reduce drinking, underscoring the need for nuanced assessment of how the event is perceived. Overall, blackout assessment may serve both a risk identification and an intervention leverage function.

Several limitations should be considered when interpreting these findings. First, blackout was assessed via self‐report, which may be influenced by recall bias; however, no alternative method of assessment for alcohol‐induced blackout currently exists. Second, the samples consisted of college students, including mandated and heavy‐drinking populations, which may limit generalizability to noncollege or older adult populations. Third, although baseline levels were statistically controlled, causal inferences regarding blackout as a turning point cannot be definitively established. Fourth, the impact of blackout may depend on subjective evaluation of the event (Fairlie et al. 2016; Merrill et al. 2019), which was not directly measured in the present work. Future research would benefit from incorporating measures not available in the current data, including alcohol tolerance, willingness to experience a blackout, and perceived severity of consequences experienced during blackout episodes, each of which may explain individual differences in whether a blackout functions as a teachable moment. Additionally, ecological momentary assessment designs, with more fine‐grained analyses than could be conducted in the current samples, could clarify short‐term behavioral adjustments (Miller and DiBello 2024) following blackout episodes and the potential reasons why. Finally, integrating physiological or event‐level drinking data may help elucidate mechanisms linking blackout to subsequent change or behavioral maintenance.

Alcohol‐induced blackouts are widely recognized as indicators of acute intoxication and elevated risk; however, the present findings suggest they may also function as pivotal events in the trajectory of alcohol involvement. Across three independent samples, baseline blackout did not predict escalating alcohol use. Instead, in two of three samples, baseline blackout was associated with reductions in alcohol‐related consequences over time. These findings suggest that blackout may, for some individuals, prompt self‐reflection or increase motivation to avoid negative consequences of drinking. At the same time, the variability across samples highlights that experiencing a blackout does not uniformly produce behavioral change—and, in all three samples, those reporting blackout at baseline maintained significantly elevated odds of future blackout occurrence. Understanding the conditions under which blackout leads to adjustment versus continued risk is an important direction for future work. Clinically, systematic assessment of blackout may offer opportunities to identify students at risk while simultaneously leveraging an intrinsically motivating experience. Rather than viewing blackout solely as an endpoint of risky drinking, it may be conceptualized as a dynamic event within the developmental course of alcohol use. Continued investigation into the dual role of blackout, as both risk marker and potential catalyst for change, may refine prevention strategies and improve intervention timing.

## Funding

This work was supported by the National Institute on Alcohol Abuse and Alcoholism (Grant No. R01AA025043, R01AA025643). NIH had no role in study design; data collection, analysis, or interpretation; manuscript preparation; or the decision to submit the paper for publication.

## Conflicts of Interest

The authors declare no conflicts of interest.

## Data Availability

The data that support the findings of this study are available from the corresponding author upon reasonable request.
